# TRPM Channels in Human Diseases

**DOI:** 10.3390/cells9122604

**Published:** 2020-12-04

**Authors:** Ivanka Jimenez, Yolanda Prado, Felipe Marchant, Carolina Otero, Felipe Eltit, Claudio Cabello-Verrugio, Oscar Cerda, Felipe Simon

**Affiliations:** 1 Faculty of Life Science, Universidad Andrés Bello, Santiago 8370186, Chile; ivankaj.dinamarca@gmail.com (I.J.); yp.prado@gmail.com (Y.P.); fe.marchant28@gmail.com (F.M.); claudio.cabello@unab.cl (C.C.-V.); 2 Millennium Nucleus of Ion Channel-Associated Diseases (MiNICAD), Universidad de Chile, Santiago 8380453, Chile; oscarcerda@uchile.cl; 3 Faculty of Medicine, School of Chemistry and Pharmacy, Universidad Andrés Bello, Santiago 8370186, Chile; maria.otero@unab.cl; 4 Vancouver Prostate Centre, Vancouver, BC V6Z 1Y6, Canada; fguersetti@prostatecentre.com; 5 Department of Urological Sciences, University of British Columbia, Vancouver, BC V6Z 1Y6, Canada; 6 Center for the Development of Nanoscience and Nanotechnology (CEDENNA), Universidad de Santiago de Chile, Santiago 7560484, Chile; 7 Millennium Institute on Immunology and Immunotherapy, Santiago 8370146, Chile; 8 Program of Cellular and Molecular Biology, Institute of Biomedical Sciences (ICBM), Faculty of Medicine, Universidad de Chile, Santiago 8380453, Chile

**Keywords:** TRPM channels, human diseases, ion channels

## Abstract

The transient receptor potential melastatin (TRPM) subfamily belongs to the TRP cation channels family. Since the first cloning of TRPM1 in 1989, tremendous progress has been made in identifying novel members of the TRPM subfamily and their functions. The TRPM subfamily is composed of eight members consisting of four six-transmembrane domain subunits, resulting in homomeric or heteromeric channels. From a structural point of view, based on the homology sequence of the coiled-coil in the C-terminus, the eight TRPM members are clustered into four groups: TRPM1/M3, M2/M8, M4/M5 and M6/M7. TRPM subfamily members have been involved in several physiological functions. However, they are also linked to diverse pathophysiological human processes. Alterations in the expression and function of TRPM subfamily ion channels might generate several human diseases including cardiovascular and neurodegenerative alterations, organ dysfunction, cancer and many other channelopathies. These effects position them as remarkable putative targets for novel diagnostic strategies, drug design and therapeutic approaches. Here, we review the current knowledge about the main characteristics of all members of the TRPM family, focusing on their actions in human diseases.

## 1. Introduction

Ion channels are proteins composed of a pore that allows for passive and regulated ion flux through biological membranes as determined by an electrochemical gradient [1,2]. Currently, more than 400 ion channels belonging to several families are known [3] and their participation has been identified in a wide range of physiological processes [4,5]. At the same time, abnormalities in the expression and function of ion channels have been associated with tissue and systemic alterations that directly contribute to the development of diseases such as cardiovascular alteration [6], organ dysfunction [7], cancer [8], neurodegenerative disorder [9] and many others channelopathies [10], which position them as interesting therapeutic targets [11].

Transient receptor potential (TRP) ion channels participate in heterogeneous physiological and pathological processes, including the regulation of vascular and immune cells [12,13,14,15], exocytosis in neuroendocrine cells [16], the differentiation of hippocampal neurons [17], the proliferation and differentiation of keratinocytes [18], hearing [19], thermosensation [20], visceral nociception [21], muscle reflex activation [22], lung function [23,24], proinflammatory cytokines production [25], dopaminergic neurons death [26], neurological development abnormalities [27], vascular permeability [28], neurogenic inflammation [29], glomerulosclerosis [30,31], hypertension [32] and cancer [33], among several others.

The first research into ion channels belonging to the TRP superfamily was carried out in 1989 by Montell and Rubin, who cloned and characterized the molecular structure of the protein encoded by Drosophila’s *trp* gene and hypothesized its possible involvement in Ca^2+^ transport during phototransduction [34]. In 1992 the first homologue, Trpl, was identified [35]. In 1993, TRP was recognized as a new ion channel superfamily and its role in Ca^2+^ permeability was determined [36]. In 1995, the first human homologue, the transient receptor potential channel-related protein 1 (TRPC1), was identified [37]. Subsequent identification and characterization of HTrp1, 2 and 3 homologues confirmed the presence of the new TRP ion channel superfamily in humans [38].

TRP channels are composed of four subunits resulting in homomeric or heteromeric channels [39]. The general structure of each subunit consists of six transmembrane helix topologies (S1–S6) where the S4 corresponds to a voltage-sensor-like domain, capable of sensing changes in intracellular ion concentration [40,41,42]. The pores of the channel are formed by a α-helix loop between the S5 and S6 subunits. Nevertheless, TRP differs from other voltage-gated channels by the aminoacidic sequence of their subunits, which confers on them differential biophysical characteristics like differences in gating probability [43], pore orientation [44] and response to different exogenous and endogenous modulators [44].

The TRP channel family is subdivided into six subfamilies based on their sequence similarity—The TRPC (canonical), TRPV (vanilloid), TRPM (melastatin), TRPP (polycystin), TRPML (mucolipin) and TRPA (ankyrin) groups [45]. The difference between TRP subfamilies is due to the high variability in length and contained domains in the N- and C-terminus (both cytosolic). The N-terminus in the TRPA, TRPC and TRPV subfamilies contains ankyrin repeats and the C-terminus in the TRPC and TRPM subfamilies contains a high conserved TRP domain [46]. Furthermore, it is well known that TRP channels activation or inhibition and the responsiveness to different modulators is strongly influenced by their large intracellular domains [44,47].

**Figure 1:**
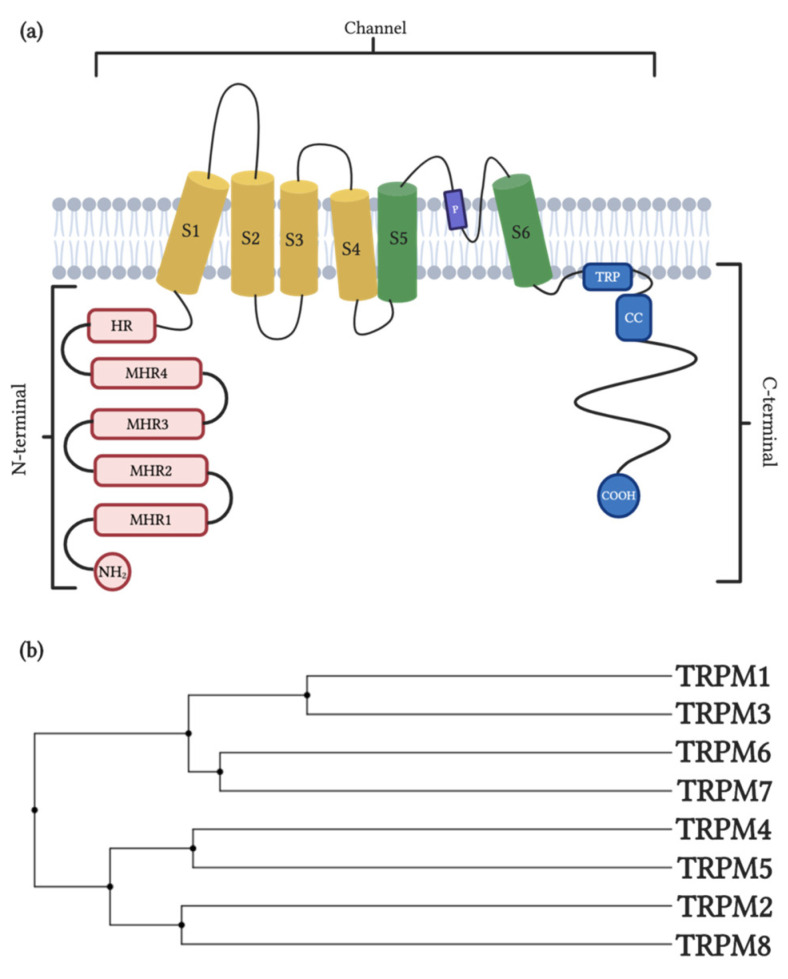
Channel structure and family tree of the transient receptor potential melastatin (TRPM) subfamily. (**a**) The N-terminus is composed of four melastatin homology regions and homology region pre-S1 (melastatin homology regions (MHR) and homology regions (HR), red boxes). The channel domain contains six transmembrane segments (S1–S6), S1–S4 (yellow cylinder) corresponding to a voltage-sensor-like domain; the pore is formed by the loop between the S5 and S6 segments (purple box and green cylinder). The C-terminus is composed of TRP and the coiled-coil (CC) (blue boxes). (**b**) Phylogeny of human TRPM channels. Created with BioRender.com.

TRPM channels have a ubiquitous expression in tissues, which is associated with participation in health and diseases [48]. These ion channels are composed of three main regions: the N-terminus, the channel domain and the C-terminus. The N-terminus is composed of 4 melastatin homology regions and one homology region or pre-S1 domain (Figure 1a, red boxes). These regions form a pocket which has been suggested to play an important role in external stimuli sensing and channel assembly [49]. The channel domain and the P-loop (Figure 1a, S1–S6 and P, respectively) are located at the transmembrane space. Mainly the S4 (but also all the S1–S4 participates, Figure 1a, yellow cylinder), is a voltage-sensing-like domain related to the activation of TRPM subfamily members. The P-loop located between S5 and S6 subunits (Figure 1a, blue loop between green cylinders) forms the ion-conducting pore [40,41,42]. Also, the C-terminus is composed of the TRP box with a highly conserved aminoacidic sequence, which is thought to play an important role in channel stability in the plasmatic membrane. Additionally, the coiled-coil domain (cc) allows for interactions between channel subunits for tetrameric complex assembling and contains specific motifs that modulate pore gating (Figure 1a, blue boxes) [50]. Three members of this family, TRPM2, TRPM6 and TRPM7, present enzymatic activity in their C-terminus. TRPM2 contains an additional nucleoside diphosphate pyrophosphatase domain which has high homology with the NUDT9-H domain [51] and TRPM6-TRPM7 contain two additional domains in the C-terminus: the serine/threonine (S/T) rich and α-kinase domain (KD) homologous with the cytosolic kinase, PLIK [52]. Based on the homology sequence of the coiled-coil in the C-terminus, the TRPM subfamily is divided into four groups: TRPM1/TRPM3, TRPM2/TRPM8, TRPM4/TRPM5 and TRPM6/TRPM7 (Figure 1b) [53]. These similarities are related to ion permeability properties, with TRPM3/TRPM6/TRPM7 being highly permeable to Ca^2+^, TRPM2/TRPM8 nonselective for monovalent and divalent cations and TRPM4/TRPM5 permeable only to monovalent cations [54].

The TRPM subfamily has gained major attention in the last years because it has been involved in several physiological and pathological processes, including cellular proliferation [55], temperature sensing [56], vascular development [57], cancer progression [58,59], neurological diseases [60], endothelial dysfunction [61] and many others. This has made the TRPM subfamily a fascinating group of ion channels with high biomedical projections. Thus, this review aims to cover an updated revision of the contribution of the TRPM ion channel subfamily in physiology and physiopathology focusing on human diseases.

## 2. TRPM1/TRPM3

### 2.1. General Properties and Distribution

Inside the TRPM subfamily, TRPM1 and TRPM3 are grouped together due to their structural similarities, sharing 75% aminoacidic identity [62]. Also, like most TRPM family members, TRPM1 and TRPM3 are non-selective Ca^2+^ channels [63]; indeed, TRPM1 is also permeable to Mn^2+^ and Mg^2+^ while TRPM3 has tissue-specific permeability to mono- and bi-valent cations associated with alternative splicing [64]. TRPM1 has a conductance of 76.70 pS [65], while TRPM3 has a conductance range between 65 to 130 pS [66,67].

TRPM1 was described for the first time by Duncan et al. (1998) in a search of loci associated with melanoma [68]. Its gene maps in chromosome 15q13.3 and is composed of 29 exons that alternatively splice to originate five isoforms that differ in the use of the 5′ exons and start codons [69,70]. The final proteins contain between 1516 and 1643 amino acids presenting a molecular weight of ~182 kDa. The TRPM1 gene encodes a microRNA (miR-211) located between exons 6 and 7 that is co-expressed with TRPM1 and is associated with diseases in the eye and skin [71]. TRPM1 was initially named melastatin. It is expressed in retina center-ON bipolar neurons and melanocytes play important roles in the melanin metabolism and signal transduction of the optic pathway [72,73].

TRPM3 was first identified in a genome library by Lee et al. (2003) [62]. Its gene, located in 9q21.12-13, is the largest in chromosome 9. It spans 870 kb including 30 exons [74]. TRPM3 has a molecular weight of between 160 and 170 kDa and contains between 1325 and 1555 amino acids. Some variants (e.g., TRPM3α1) are selective to monovalent cations, while others (e.g., TRPM3α2) are highly selective for divalent cations [75]. The *trpm3* gene contains large intronic sequences between exons 1–2 and 2–3 that contain recognition sites for transcription factors [76]. Related to this, it has been shown that the *trpm3* gene encodes an intronic microRNA (miR-204) that is transcribed with TRPM3 [77]. The encoded protein has been proposed to have at least 23 splice variants [78] but only a few of them have been found in tissues. TRPM3 is expressed mostly in nociceptive neurons, pancreatic beta cells, the kidney and the vasculature muscular layer but has also been described in various parts of the brain, the ovary, the prostate, odontoblasts, adipocytes, the oral mucosa, the ciliary body and the retinal pigmented epithelium [66,79,80,81,82,83]

### 2.2. Activation and Inhibition: Endogenous Modulators

Both channels are not voltage-gated and respond to a variety of physical and chemical stimuli. TRPM1 is activated in bipolar retinal neurons by glutamate decrease [65]. Although its activation outside of the retina remains mostly enigmatic, factors such as UVB light enhance TRPM1 expression in skin, inducing melanocyte migration and pigmentation [84]. TRPM3 is activated by physical events such as heat or osmolarity, as well as sphingolipids [66,85].

Also, several transcription factors enhance TRPM1 and TRPM3 expression. Microphthalmia-associated transcription factor (MITF) has been reported as a transcription factor that regulates TRPM1 and TRPM3 expression in the eye [86,87,88]. In the retina, the basic helix-loop-helix transcription factor b4 (Bhlhb4) has been suggested as a transcription factor for TRPM1, as the bHLHb4 mutant presents downregulation of TRPM1 [89]. TRPM3 expression is promoted by Pax6 during lens development in fish and humans and is inhibited by transcription factor STAT3 [78,90].

**Figure 2:**
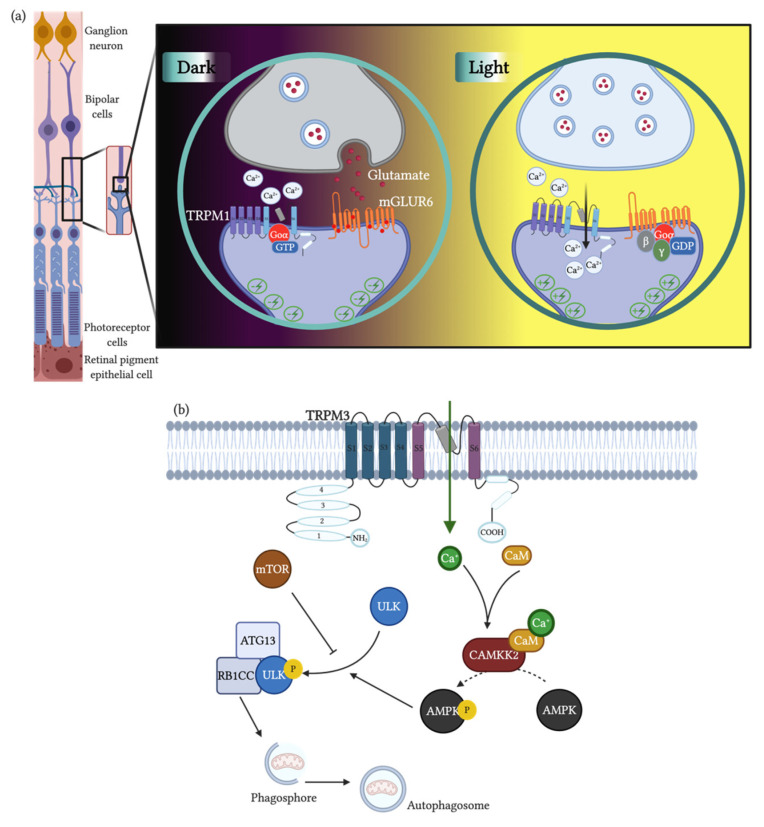
TRPM1 role in visual pathways. In the retina, the photoreceptors are in synaptic contact with bipolar neurons. (**a**), which in turn contact ganglion neurons, in which axons merge to form the optic nerve. In the dark, the rods secrete glutamate into the synaptic cleft, which activates the mGluR6, a G-protein coupled receptor. In its activated state, mGluR6 hyperpolarizes the neuron. In that status, G-protein Goα-GTP is bound to TRPM1, resulting in inactivation of the channel. In the presence of light, rods release low glutamate, which inactivates mGluR6. As a consequence, the dephosphorylation of GTP into GDP allows Go to bind and subunits and the complex binds mGluR6, inactivating it and avoiding hyperpolarization. The release of Go activates TRPM1, which opens, allowing calcium entry and depolarizing the bipolar neuron triggering the electrochemical signaling. (**b**) TRPM3 and autophagy in clear cell carcinoma. TRPM3 activity increases cytosolic calcium, which binds Calmodulin (CaM). Ca-CaM binds CaMKK2 and this activated complex phosphorylates AMPK. Active AMPK through phosphorylation, in turn, phosphorylates ULK, which, in its phosphorylated form, can bind ATG13 and RB1CC1. This ULK-ATG13-RB1CC1 complex allows for the formation of autophagosomes and the binding of the autophagosomes with lysosomes. ULK is normally inactive through the phosphatase activity of MTOR. Thus, in normal conditions, this pathway is inactive. Created with BioRender.com.

TRPM1 activity has been well described in the retina, where it participates in On-center bipolar cell transduction. In mammals, photoreceptors hyperpolarize in response to light [91]. They release glutamate as a neurotransmitter in direct proportion to their depolarization status. Thus, under light, they release less glutamate, while in the dark they release higher levels of glutamate [92]. On-center bipolar cells (post-synaptic) express the metabotropic receptor mGluR6 (G-protein coupled), which hyperpolarizes the cell in the presence of glutamate, as happens in the dark [92]. In the light, with lower levels of glutamate, the mGluR6 receptors do not hyperpolarize the On-center bipolar cells. Consequently, TRPM1 channels open in the synaptic cleft, allowing the entry of Ca^2+^ to the postsynaptic cell and the subsequent continuation of the electrochemical wave [65]. This mechanism is critical in rod transmission and thus to light sensitivity in mammals (Figure 2a).

A particular characteristic of TRPM3 is its constitutively basal activity, which could be increased or decreased by a variety of substances [66]. Unlike other TRPMs are activated by sphingosine-1 sulphate, a signaling sphingolipid that modulates vascular and immune systems [93]. Pregnenolone sulphate is an excitatory neurosteroid that activates TRPM3 channels. TRPM3 activation increases cytosolic calcium, which activates Ca/calmodulin, which in turn triggers signaling through mitogen-activated protein kinases (MAPKs) [78]. This influx of Ca^2+^ also activates c-Jun N-terminus protein kinases 1 and 2 (JNK1/2) [94]. The final result of the activations of these pathways is the targeting of specific transcription factors that would modulate gene expression. It has been demonstrated that AP-1 and ERG-1 are transcription factors that are the target of TRPM3 activation by pregnenolone sulphate; however, nifedipine and D-erytro-sphingosine (and TRPM3 activators) do not generate such an effect [95]. In pancreatic cells, the activation of the MAPKs pathways transcription factor ERK is enhanced by the TRPM3-dependent activation of ERG-1. Activated ERG-1 binds to regulatory regions of Pdx-1, a major regulator of insulin expression [96]. TRPM3 is also activated by hypotonicity (200 mOsm/L), which, together with its expression in the kidney, suggests that TRPM3 plays a role in Ca^2+^ homeostasis [66]. Steroids such as cholesterol and steroidal hormones partially block TRPM3; however, the mechanisms and physiologic impact of this inhibition are unclear [97]. Recently, the inhibition of TRPM3 by G protein βγ subunits released from Gi/o, Gs or Gq coupled receptors has been demonstrated [98,99]. In mouse dorsal root ganglion (DRG) neurons], the inhibition of TRPM3 by the Gi-coupled GABA-B receptors mediated by G protein βγ subunits is thought to be a regulatory mechanism of nociceptive response [100] and it is suggested to be a main mechanism of action of opioids in analgesic treatment [101].

Both TRPM1 and TRPM3 are inhibited by intracellular bivalent cations, while Zn^2+^ effectively inhibits TRPM1 activity and Mg^2+^ inhibits TRPM3 [102]. The activation of TRPM1 and TRPM3 has a dual effect. On the one hand, the entry of cations depolarizes the cells or changes their chemical-electric status. On the other hand, through second messengers, they activate transcription factors, affecting gene expression.

### 2.3. Physiological Function and Role in Diseases

Importantly, both are associated with several human diseases by either their increased or decreased expression or activity. Together with the previously described role in the retina, TRPM1 expression is associated with melanocyte activity and differentiation. Reduced expression of TRPM1 is associated with lower melanin synthesis [103] and its loss of expression is a prognosis marker of primary melanoma [68,73,104]. The similarities in expression of TRPM1 in melanocytes and neurons are not surprising because melanocytes are derived from the neural crest. Indeed melanocytes, similar to bipolar ganglionic cells, express mGluR6, which in melanocytes enhances the activity of TRPM1 [103]. The expression of TRPM1 in the retina and skin is perhaps best observed in miniature horses with congenital stationary blindness. The visual disease in this specie is accompanied by a leopard complex of the skin (dark dotted pattern), caused by mutations in *trpm1* [105]. Although retinal and melanocyte pathologies are the best-known diseases in which TRPM1 is involved, recently through SNP analysis, *trpm1* was defined as a locus that confers susceptibility to coronary artery disease, although the pathogenic mechanisms have not been described [106].

The best-described function of TRPM3 is its role in noxious heat detection and consequent heat-associated inflammation [107]. Increased temperature increases cytosolic Ca^2+^ by TRPM3 activation [108] in what is thought to be a heat-sensing system in nociceptive neurons in conjunction with other ionic channels, such as TRPV1 and TRPA1 [109]. However, triple-negative mice (TRPV1^–/–^, TRPA1^–/–^ and TRPM3^–/–^) can still respond to temperature changes, suggesting that other channels and proteins are involved in thermo perception [110]. Recent studies have demonstrated that TRPM3 activation triggers the secretion of Interleukin 8 via the activation of c-Jun and activating transcription factor 2 (ATF2), which helps to explain the inflammatory changes associated with TRPM3 activation [111]. In arteries, the activity of TRPM3 was first described to contract a mouse aorta through its effects on smooth muscle [97]. Conversely, in mesenteric arteries, TRPM3 is restricted to sensory nerve endings and its activation dilates mesenteric arteries [112]. Also, mutations in *trpm3* have been associated with different modalities of glaucoma and cataract [113,114]. Pediatric cataract with autosomic dominant transmission was the first disease to be associated with *trpm3*, and TRPM3/miR-204 has been proposed as an important complex in eye development [78,114]. In the endocrine pancreas, the activation of TRPM3 channels increases insulin secretion mediated by an increase of cytosolic Ca^2+^ and cell depolarization [108,115]. However, the effect of this activation is controversial because *trpm3*^−/−^ mutant mice have normal values of glucose with no signs of metabolic disorders [116]. More recently, two mutations in *trpm3* that generate an overreactive channel were associated with epilepsy and intellectual disability as a result of increased intracellular Ca^2+^ in the brain [117,118,119].

Recent studies have demonstrated the implication of TRPM3 and miR-204 in the pathogenesis of clear cell renal cell carcinoma (ccRCC) by affecting autophagy, which is a critical event in ccRCC [120,121]. Specifically, TRPM3 plays a major role in the progression of ccRCC with von Hippel-Lindau (VHL) loss mutation and has an increased expression in human ccRCC with inactivated or deleted *vhl* [122]. VHL is a ubiquitin ligase that inactivates hypoxia-inducible factor (HIF) activity. When VHL is lost, the cytoplasmic levels of HIF increase and cells undergo a state of pseudohypoxia triggering autophagy [123].

Autophagy is a process by which cells remove dysfunctional components allowing for the degradation and recycling of components, enabling cell survival in hypoxia, stress or nutrient deficiency [124]. In cancer, autophagy is a double sword mechanism. On the one hand, autophagy is associated with a reduction of metastatic potential but on the other hand, it promotes cell survival in hypoxic areas of the tumor [121]. In clear cell carcinoma, an “autophagic-switch” promotes autophagy enhancing cell survival, being a target in cancer therapy [125]. Autophagy starts with the formation of an autophagosome. This process is controlled by ULK1, a protein that allows for the formation of the phagosome and its fusion with the lysosomes that contain the enzymes for degradation [126]. ULK1 is part of a complex with ATG13 and RB1CC1 and is usually inactivated by mTOR. On the opposite side, AMPK is an activator of ULK1 and its activity results in increased autophagy [127]. Simultaneously, the activation of AMPK inhibits mTOR. Here, TRPM3 activity increases cytosolic Ca^2+^ that binds to Calmodulin, subsequently activating the Ca/Calmodulin Dependent Protein Kinase Kinase-2 (CAMKK2), which phosphorylates and activates AMPK [128] (Figure 2b). This effect of TRPM3 and autophagy in ccRCC has been confirmed by the effects of mefenamic acid, a non-steroidal anti-inflammatory that was described as a specific blocker of TRPM3 [129]. After daily doses of mefenamic acid, mouse xenografts of ccRCC stop growing or even experience tumor regression [130]. The role of miR-204 is antagonic to TRPM3, as miR-204 directly inhibits TRPM3 translation by binding the 3′ UTR region of the mRNA and has an indirect effect by inhibiting the translation of caveolin1, a structural component of membrane caveoli, which is necessary for TRPM3 expression [131].

Main TRPM1/TRPM3 endogenous and exogenous modulators, participation in diseases and physiological functions are listed in Table 1.

## 3. TRPM2/TRPM8

### 3.1. General Properties and Distribution

TRPM2, also named TRPC7 or LTRPC2 and TRPM8 [141,142] are Ca^2+^ permeable cation channels with rather low Ca^2+^ selectivity [143,144]. Specifically, TRPM2 is permeable to Na^+^, K^+^, Ba^2+^, Ca^2+^ and Mg^2+^, with relative permeability to these ions described as P_K_/P_Na_—1.1, P_Ca_/P_Na_—0.9 and P_Mg_/P_Na_—0.5 and similar ratios for Ca^2+^, Mg^2+^ and Ba^2^ [145,146]. On the other hand, TRPM8 has relative permeability to Ca^2+^, which varies between 0.97 and 3.2, to monovalent ions. The permeability order is Cs^+^ > K^+^ > Na^+^, while for divalent cations it is Ba^2+^  > Ca^2+  ^>  Mg^2+^ [145].

Under physiological conditions, TRPM2 single-channel conductance range is between 52 to 80 pS with unusually long open times in the range of several seconds [67]. On the other hand, TRPM8 single-channel conductance depends on the temperature, being 60 pS at 10 °C and 75 pS at 30 °C [51,147,148]. Moreover, it was shown that when sensors are fully activated by voltage, the absolute open probability value is less than one and that this value decreases when the temperature rises, suggesting that TRPM8 is also partially activated by voltage in a temperature-dependent manner [149]. TRPM2 is highly expressed in the brain, heart, spleen, liver and lung, as well as in different cell types, such as immune cells (including monocytes, neutrophils and macrophages) [150,151], whereas TRPM8 is mainly expressed in the prostate and liver [48,80,141,152,153,154].

Complete structures of TRPM2 and TRPM8 have been determined by cryo-electron microscopy, providing information related to their channel assembly, gating mechanisms and structural pharmacology [63,155]. TRPM2 is known as a channel/enzyme protein, meaning that it is an ion channel that possesses an enzymatic region, based on its C-terminus Nudix domain (NUDT9-H) homologous to the NUDT9 adenosine diphosphate ribose (ADPR) pyrophosphatase. This enzyme converts ADPR to adenosine monophosphate and ribose 5-phosphate [51,156].

Despite the lack of enzymatic regions in TRPM8, the study of its structure has shown the importance of the classical protein domains for channel function. For instance, studies by Phelps and colleagues through electrophysiology and microscopy analysis show that amino acids 40 to 86 within the melastatin homology region 1 (MHR1) (N-terminus) are essential for TRPM8 localization in the plasmatic membrane [157]. The coiled-coil domain (C-terminus) of TRPM8 modulates channel maturation and trafficking to the plasmatic membrane and is required for TRPM8 subunit interaction for tetramer pore assembly [158].

### 3.2. Activation and Inhibition: Endogenous Modulators

The NUDT9-H domain of TRPM2 mediates ADPR binding to the channel and is required for ADP-ribose-dependent channel activation [159,160]. Likewise, intracellular Ca^2+^ and arachidonic acid potentiate channel activation by ADPR, where the Ca^2+^ influx through the channel provides positive feedback that enhances TRPM2 activation [161,162]. Moreover, this channel is also known as a redox-sensitive cation channel, as it promotes Ca^2+^ influx after activation by reactive oxygen species (ROS) through poly(ADP-ribose) polymerase-ADPR-dependent (PARP) and also responds to reactive nitrogen species (RNS), releasing ADPR from mitochondria and the overproduction of TNF-α [60,162]. It also inhibits ROS production in phagocytic cells and endotoxin-induced lung inflammation in mice [163].

**Figure 3:**
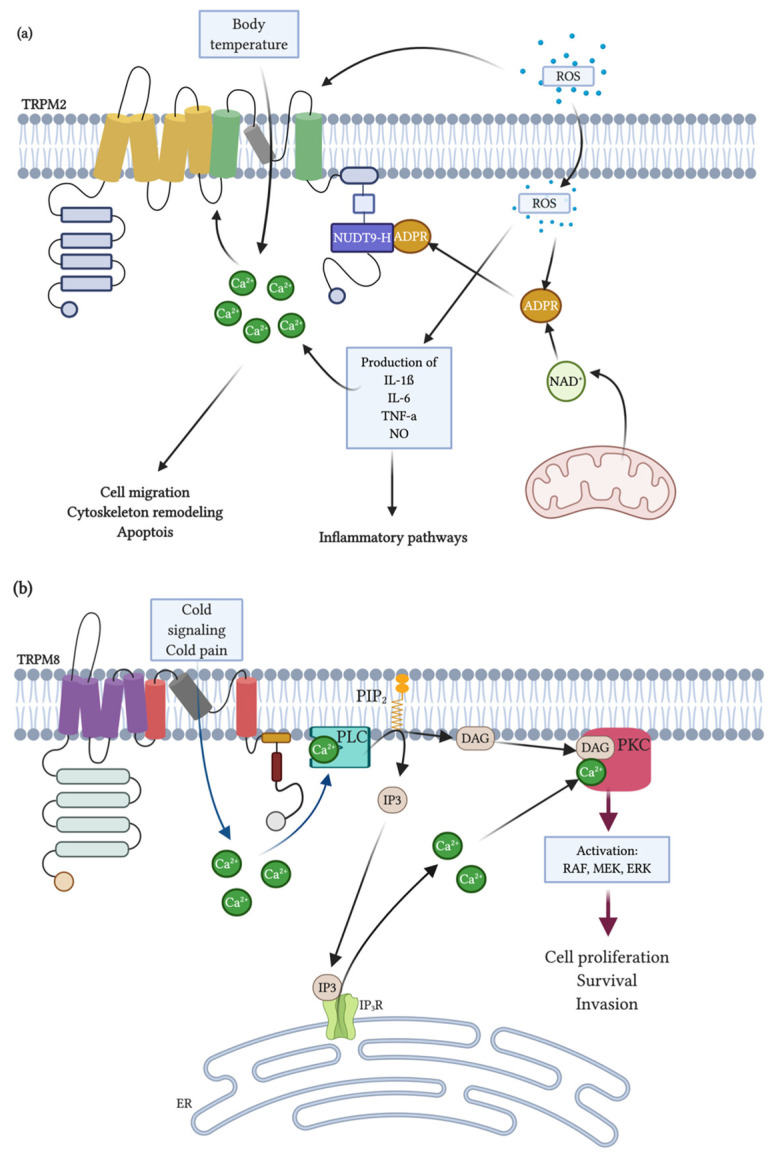
Signaling mechanisms for TRPM2 and TRPM8 activation. (**a**) NAD+ and reactive oxygen species (ROS), including H_2_O_2_, accumulate during inflammation and tissue damage may trigger TRPM2. NAD+ may be converted to ADPR and cADPR. ROS can also cross the plasma membrane and mobilize ADPR from mitochondria and both ROS and cADPR can synergize with ADPR to activate TRPM2. Additionally, ADPR is generated from NAD+ via poly-ADPR during ROS-induced damage through the activation of PARP (poly(ADP-ribose) polymerase)-ADPR-dependent mechanisms in inflammatory cells. Free cytosolic ADPR can act on the NUDT9-H of TRPM2 channels, enabling Ca^2+^ influx across the plasma membrane and/or release of intracellular Ca^2+^, raising the Ca^2+^ concentration in the cytosol. On the other hand, ROS induces cytokine production (pro-inflammatory response), which may alter intracellular calcium levels. A Ca^2+^ increase will activate different physiological processes including gene expression through Ca^2+^-dependent signaling pathways such as apoptosis, cell migration and cytoskeleton remodeling. Moreover, TRPM2 may detect increased temperatures to prevent overheating, limiting the fever response. (**b**) Cold temperature, menthol or icilin can stimulate the activity of the TRPM8 ion channels. Upon activation, the TRPM8 channel changes conformation and permits extracellular Ca^2+^ to flow through it across membranes. This results in the activation of Ca^2+^-sensitive PLC and the hydrolysis of PIP_2_, which produces inositol 1,4,5-triphosphate (IP_3_) that causes the release of Ca^2+^ from the intracellular stores and generates diacylglycerol (DAG). The elevated Ca^2+^ level or DAG can activate protein kinase C (PKC), which, in turn, activates RAF in the ERK pathway. Therefore, transcriptional activation of genes stimulates cellular proliferation, survival and invasion, which contribute to cancer growth and metastasis. Created with BioRender.com.

Furthermore, it has been shown that TRPM2 is also activated by ADPR structural analogues, like cADPR, nicotinic acid adenine dinucleotide phosphate (NAADP) and 2′-O-acetyl-ADP-ribose (OAADPr), a product of the sirtuin family of deacetylases proteins [164]. Furthermore, intracellular Ca^2+^ and ADPR likely act co-operatively to induce TRPM2 channel activation (ADP-ribose acts as a second messenger through its ability to gate TRPM2, leading to Ca^2+^ entry). Such co-operativity between ADPR and Ca^2+^ likely has important functional implications. In neutrophils, endogenous ADPR levels are sufficient to allow TRPM2 to respond to varying intracellular levels of Ca^2+^ released from intracellular stores [165]. These results indicate that TRPM2 integrates intracellular signaling events, modulating changes in ADPR and Ca^2+^ intracellular concentrations [60,166] (Figure 3).

Then, Ca^2+^, cADPR, H_2_O_2_ and NAADP positively modulate TRPM2, whereas AMP and acidic pH negatively regulate it [167]. Moreover, there are several known TRPM2 inhibitors, including *N*-(*p*-amycinnamoyl) anthranilic acid (N-ACA), econazole, flufenamic acid, clotrimazole, 2-aminoethyl diphenylborinate (2-APB), anthranilic acid and curcumin [168,169,170,171,172,173,174]. However, most compounds lack either specificity and/or potency. Nevertheless, another inhibitor has been characterized in a Cacospongia extract, which efficiently inhibits TRPM2-mediated currents in a concentration and time-dependent manner. Starkus and colleagues described that the sesterterpenes scalaradial and 12-deacetylscalaradial molecules contained in this extract might act as potent TRPM2 inhibitors [175]. Also, inhibitors of poly (ADP-ribose) polymerase, a potential enzymatic source of ADPR, prevent TRPM2 activation but do not directly block the channel [176].

For TRPM8 activation, the prominent voltage-dependent gating properties are activated by cold temperature [141,154] and by natural and synthetic “cooling” agents such as menthol (a cyclic terpene alcohol extracted from peppermint leaves, *Mentha piperita*), eucalyptol (a natural organic compound derived from the *Eucalyptus* tree) and icilin (a synthetic cooling compound) [177,178,179]. Furthermore, it has been established that the most selective TRPM8 ligands are menthol derivates (CPS-368, CPS-369, CPS-125, WS-5 and WS-12). The selectivity and improved activity seem to be due to the hexacyclic ring structure present in all these compounds. Conversely, agents such as WS-23 that lack this functional group, weakly activate TRPM8 and substances with a pentacyclic ring structure maintain TRPM8 inactivity [180].

Like other TRPM channel family members, TRPM8 requires PIP_2_ for its activation, with specific R and K residues in the TRP domain being essential for PIP_2_-dependent channel gating [181,182].

Concerning TRPM8 inhibitor effects, it has been shown that they remarkably reduced cold and mechanical allodynia in acute and chronic pain models [183]. Specifically, IGM-18 administration reduces body temperature in a dose-dependent manner, suggesting that the reduction of pain is due to the modulation of pain pathways, without any alteration of body temperature [184]. Furthermore, another approach to TRPM8 antagonism has been developed through compounds that inhibit channel-induced gene transcription, analyzing whether a particular compound interrupted the signaling cascade connecting TRPM8 channel activation (by either icilin or menthol) with the activation of the transcription factor AP-1. Then, it was demonstrated that the compounds RQ-00203078, BCTC, TC-1 2014, 2-APB and clotrimazole inhibited TRPM8-induced activation of AP-1 but also attenuated transcriptional induction mediated by stimulation of either TRPM3 and/or TRPV1 channels. Nevertheless, while most compounds function as broad-spectrum Ca^2+^ channel inhibitors, only the RQ-00203078 compound showed specificity for TRPM8 [183,185,186].

### 3.3. Physiological Function and Role in Diseases

TRPM2 is implicated in different cellular and physiological processes, including cytokine production, cell death, oxidative stress response and fibrosis. Moreover, TRPM2 is an essential factor in cell death induced by oxidative stress through the activation of caspase cleavage [187,188].

As an identified nonselective Ca^2+^-permeable cation channel and the sensor of ROS (causing cell damage by elevating intracellular calcium content under oxidative stress), TRPM2 has been recently demonstrated as being involved in the unilateral ureteral obstruction (UUO)-triggered renal fibrosis. Related to this, it was shown that TRPM2 activity is involved in renal fibrosis through JNK pathway activation [189]. Also, the absence of TRPM2 triggered less production of inflammatory mediators and decreased apoptosis-related protein expressions in response to lipopolysaccharide (LPS)-induced sepsis [166]. Moreover, other studies in hippocampal neurons indicate that TRPM2 deletion might regulate inflammatory response [163,166,190]. These findings were confirmed in an Alzheimer’s mouse model showing that TRPM2 plays an important role in β-amyloid-mediated neuronal toxicity and memory impairment [191].

The TRPM2 channel is also found in glial cells (microglia and astrocytes) and plays an essential role in pathophysiological disorders [192]. Furthermore, recent studies have suggested that TRPM2 inhibition is effective for preventing ischemic acute kidney injury by relieving oxidative stress, inflammation and apoptosis [193]. Hence, the TRPM2 channel is an essential regulator of plasticity [194,195]. In addition, it has recently been described that this ion channel might also behave as a temperature sensor in a subpopulation of hypothalamic neurons. Then, TRPM2 can detect increased body temperature to prevent overheating, thereby restricting the fever response [196].

On the other hand, the TRPM8 channel plays a critical role in cold perception [179]. This channel is, then, the principal molecular transducer of cold somatosensation, in a range between 8–25 °C [197]. In terms of pain, it is reported that inhibition of TRPM8 correlates with a decrease in acute and chronic symptoms; there are also results of analgesia (especially in inflammatory and neuropathic conditions) in the activation of TRPM8 [184].

In some pain-related disorders, such as osteoarthritis, the activation of peripheral receptors that express TRPM8 has analgesic characteristics [198,199]. Cooling the skin can produce a calming sensation on the inflammatory pain. Also, natural TRPM8 agonists, such as menthol, have been used for centuries due to their analgesic, antipruritic and counterirritant effects [200,201]. Accordingly, it has been demonstrated that cold chemical agonists have analgesic properties. Therefore, the participation of this channel in pain modulation has been largely investigated [177,184]. Then, inflammation and nerve injury might end in cold hypersensitivity, mediated by TRPM8 activation. Thus, cold hypersensitivity treatment may use compounds that inhibit activation of the TRPM8 channel. Then, apart from its role in cold perception, this channel contributes to cold allodynia after inflammation or nerve injury, which makes it is essential for cooling/menthol-based analgesia [202].

Furthermore, the TRPM8 channel is one of the most promising clinical targets for the metastatic transition of prostate cancer (PC). This fact is based on several studies proposing that this channel plays a key role in the regulation of PC cell migration [152,203]. Its expression increases during the initial stages of PC but is reduced after anti-androgen therapy [204]. Consequently, it has been described that TRPM8 gene regulation would be androgen-dependent [205].

Interestingly, several groups have reported the role of TRPM8 in prostate cancer progression. Yang and colleagues observed that the overexpression of TRPM8 inhibits migration and proliferation of the androgen-independent prostate cancer cell line PC-3, also facilitating starvation-induced apoptosis [206]. In the context of migration, it was reported that TRPM8 is capable of inhibiting endothelial cell migration via Rap1 GTPase, by direct intracellular interaction between both proteins [207]. Furthermore, nanoparticles loaded with the TRPM8 agonist W12 inhibit the proliferation and migration of PC-3 cells through the activation of the TRPM8 channel, in both in vitro and in vivo models [208]. More recently, it was demonstrated that androgen-dependent inactivation of TRPM8 enhances migration of prostate cancer cells, demonstrating the existence of a non-genomic mechanisms involved in the regulation of the TRPM8 channel by androgens and the role of TRPM8 in the inhibition of cancer cell migration [205].

Investigations performed on mice deficient in thermosensitive transient receptors exposed that temperature-dependent screening was impaired in these two mice models (Trpm2- and Trpm8-null mice) [196]. Moreover, a TRPM2-genetically deficient mouse has been developed in different inflammatory models, revealing the involvement of TRPM2 in various innate immunity properties [209]. Consequently, the absence of the TRPM2 channel-mediated function resulted in increased inflammation under *L. monocytogenes* infection as compared to WT mice [210]. Also, it has been shown that this mice model is largely protected from dextran sulfate sodium (DSS)-mediated colitis [211].

The analysis of TRPM8-deficient mice confirmed that TRPM8 channels play a fundamental role in thermosensation [212]. Then, TRPM8-deficient mice were not able to detect innocuous cold temperatures and display a partially defective phenotype in responding to noxious cold [213,214]. Moreover, through this model, the role of TRPM8 in regulating pain perception has been extensively investigated [214,215].

Therapeutically, the TRPM2 inhibitors described above have revealed a lack of specificity resulting in a significant limitation. However, in the last five years, research has developed some innovative TRPM2 inhibitors. These compounds showed an effect in experimental data, with some of them promising in terms of development into novel therapeutics [166]. For instance, it has been shown that in mouse neutrophils and dendritic cells, the use of an ADPR modified analog (8Br-ADPR) could inhibit calcium influx triggered by ADPR [216]. After this positive experience, many other compounds were designed and their TRPM2 antagonism capacity was assessed, that is, the antagonist 8-Ph-2′-deoxy-ADPR [166]. Moreover, a novel ADPR analog has been synthesized which, at low concentrations, inhibits currents produced by TRPM2 activation in a specific manner without disturbing other TRPM channels in an in vitro model [217]. Additionally, experiments based on tat-M2NX, a cell-permeable peptide (a TRPM2 peptide inhibitor), which has been recently synthesized to interact with the C-terminus NUDT9-H domain, showed a calcium influx decrease providing protection from ischemic stroke in young adult and aged male animals with a clinically relevant therapeutic window [60,218]. On the other hand, other studies have found that TRPM2 suppression (which reduces inflammation and renal fibrosis by blocking TGF-β1-regulated JNK activation) might be a possible therapeutic target in renal fibrosis and chronic kidney disease prevention [189].

Far beyond its function as a thermoreceptor, since its cloning almost 20 years ago, diverse research on TRPM8 pathological and neurophysiological roles has been developed, with TRPM8 identified as a potential target to reduce symptoms or cure many disorders. Supported by the scientific literature, Axalbion’s in vivo and ex vivo proof of concept experiments suggests that its lead candidates, AX-8 and AX-10, could be used to treat diseases such as chronic cough and dry eyes (© 2020 Axalbion).

Main TRPM2/TRPM8 endogenous and exogenous modulators, participation in diseases and physiological functions are listed in Table 2.

## 4. TRPM4/TRPM5

### 4.1. General Properties and Distribution

TRPM4 and TRPM5 are channels permeable to monovalent cations and, under physiological conditions, their single-channel conductance range between 23 to 25 and 16 to 25 pS, respectively [67,227,228,229,230,231]. In particular, TRPM4 has a conductance preference for Na^+^ > K^+^ > Cs^+^ > Li^+^ >> Ca^2+^, Cl^−^ and TRPM5 for Na^+^ ≥ K^+^ ≥ Cs^+^ > Li^+^ [232].

The TRPM4 gene is encoded on chromosome 19 and its protein product contains 1213 amino acids, whereas TRPM5 is an 1165 amino acid protein encoded on chromosome 11 [233]. These channels present a transmembrane domain (TMD) composed of 6 α-helix motif (S1 to S6) [231], between S5 and S6 is the pore region, which includes the residues R964-R965 and S970 highly conserved in the TRP family (except for TRPM2), which is important for channel regulation [231,234]. At the intracellular N-terminus are four domains (MHR1 to MHR4), highly conserved in the TRP family [231]. Both the MHR3 and MHR4 domains interact with the TMD through MHR4. In this region, MHR1 also interacts with the MHR3 domain, forming a complex incapable of interacting with the central coiled-coil (CH3). Finally, at the intracellular C-terminus are the coiled-coil domain (CH1-CH3), composed of three alpha-helix domains and which interacts with a large number of phospholipids on the inside of the plasma membrane. This interaction gives rigidity to the pore structure [231]. Unfortunately, no detailed studies describe the TRPM5 structure [229].

It has been shown that TRPM4 is expressed in different tissues, with predominance in the intestine and prostate [48,80]. Also, this channel is expressed in cells from innate and adaptive immune response [41,230,235]. The TRPM5 expression is more specific than TRPM4 and is restricted to intestine, pancreas, prostate and taste cells [48,80,233].

### 4.2. Activation and Inhibition: Endogenous Modulators

**Figure 4:**
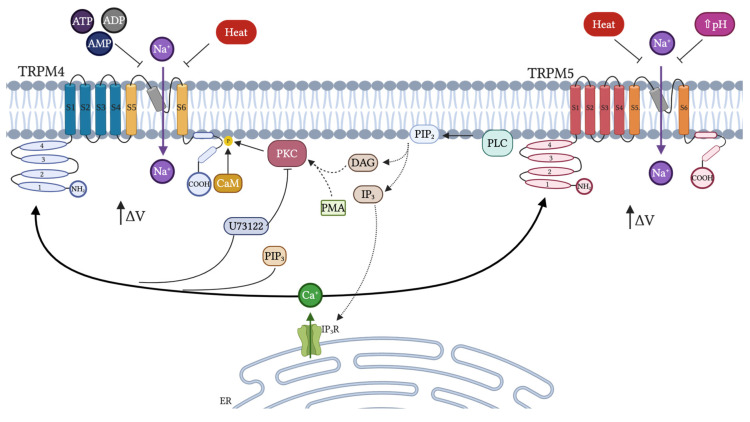
Endogenous regulation of TRPM4 and TRPM5. Both TRPM4 and TRPM5 are activated by [Ca^2+^]i and regulated by PIP2. TRPM4 also is activated by CaM and PKC by phosphorylation of the TRP region. PLC is fundamental for the regulation of both channels due to the activation of PKC via DAG and also mediates the release of calcium from ER via IP3. Finally, these channels are inhibited very differently. In the case of TRPM4, it is inhibited by AMP, ADP and ATP, while TRPM5 is inhibited by a high pH or heat. Created with BioRender.com.

Like many members of the TRP family, TRPM4 and TRPM5 are regulated by phosphatidylinositol (4,5)-bisphosphate (PIP_2_) [41,230,236]. In recent years, it has been demonstrated that amino acids R755 and R767 in the N-terminus of TRPM4 are important for direct interaction with PIP_2_ and phosphatidylinositol (3,4,5)-trisphosphate (PIP_3_) ligands [237]. Unlike other melastatine subfamily members, these channels are activated by a rise in [Ca^2+^]i and it has been shown that TRPM5 is 5–10 times more sensitive than TRPM4 [232]. TRPM4 and TRPM5 also respond to external stimulation by ATP [41,228,230]. Related to this, Nilius and colleagues demonstrated that TRPM4 is sensitive to different adenine nucleotides in the order ADP > ATP > AMP >> adenosine and could be inhibited by 1 μM ATP, while TRPM5 remained insensitive (Figure 4) [232,238]. Another important regulatory difference between TRPM4 and TRPM5 was exposed using U73122, a PKC inhibitor [239]. U73122 was able to modulate the activity of TRPM3 and TRPM4 but not of TRPM5, in which the activation of TRPM4 by U73122 was independent of PIP_2_ and Ca^2+^ [240]. These differences in endogenous regulation between TRPM4 and TRPM5 are explained by their structural differences, as most of these cellular processes occur due to the physical interaction of channel domains with regulatory molecules and subsequent conformational change in the channel structure. Interestingly, calmodulin (CaM) was shown to regulate the sensitivity of TRPM4 to Ca^2+^, as, using CaM negative mutants, TRPM4 decreases its activation. In addition, mutations in the C-terminus binding site of CaM result in a reduction in the amplitude of the current and promote faster decay [235].

TRPM4 and TRPM5 activity is also regulated by heat, which has been observed to change the voltage-dependent activation curve [241]. However, none of these channels are considered real thermal receptors.

On the other hand, it has been shown that TRPM5 presents an [IC_50_] at a pH of 6.2 and a total block at pH 5.9. This was demonstrated by studying a double TRPM5 mutant for E830 (S3-S4 linker) and H934 residues (S5-S6 linker), which turned out to be extremely insensitive to extracellular pH. Interestingly, these residues are not conserved in TRPM4 (Figure 4) [242].

The first exogenous inhibitors identified were flufenamic acid and spermine, both non-specific inhibitors which also inhibit TRPM4 and TRPM5 [232,238,243]. Another TRPM4 inhibitor is 9-phenanthrol, which has been reported to exhibit a certain specificity for TRPM4, producing a decrease in its activity [244,245]. Also, glibenclamide has been reported as a TRPM4 inhibitor, which blocks TRPM4 current in the sinoatrial node cell at a concentration of 100 μM In recent years, the compound 2-chloro-2-(2-chlorophenoxy) acetamido benzoic acid (CBA) has been reported as a powerful TRPM4 inhibitor [246,247].

### 4.3. Physiological Function and Role in Diseases

The first indications of a pathophysiological role for TRPM4 were provided by Earley and colleagues, reporting that its downregulated expression attenuated smooth muscle cell depolarization. Also, it was observed that the myogenic vasoconstriction of cerebral arteries decreased, demonstrating that the decrease in TRPM4 activity affects blood flow in the brain [248]. Later, using an ex vivo study model, it was observed that TRPM4 is an important link between the activation of the P2Y mechanoreceptor and the myogenic constriction of cerebral parenchymal arterioles (PAs) isolated from the brains of rats. TRPM4 inhibition by 9-phenanthrol provoked a strong decrease of the myogenic tone of isolated PAs. Congruently, the downregulation of TRPM4 using antisense oligodeoxynucleotide (AO) decreases the vasoconstriction induced by the specific ligands UTPγS and UDP [249].

Afterwards, Gerzanich and collaborators, using rodent models of spinal cord injury (SCI), demonstrated the importance of TRPM4 in the progressive expansion of the second hemorrhage associated with vascular fragility. Suppression of TRPM4 by AO in rats after SCI maintains the integrity of the capillary structure and generates an increase in neurological function [250], supporting its pathological role in the vasculature. Also, evidence has emerged supporting the notion that TRPM4 participates in modifying endothelial structure and function, due to its inhibition using drugs and short interfering RNA and promotes TGF-β-mediated fibrotic conversion of endothelium cells (EC), demonstrating its role in endothelial transformation under inflammation and suggesting that TRPM4 could participate in the Ca^2+^ signaling required for endothelial fibrosis [251]. Interestingly, it has been shown that the LPS-induced endothelial transformation process can also be mediated by TRPM7, suggesting potential crosstalk between TRPM4 and TRPM7 [251,252].

Inhibition of TRPM4 in mouse embryonic fibroblasts (MEFs) results in reduced cellular spreading, migration and contractile behavior and impacts the turnover of focal adhesions, serum-induced Ca^2+^ influx, FAK and Rac activities. This effect was also observed in an in vivo model of skin wound healing [253]. Interestingly, TRPM4 was shown to be crucial in LPS-induced endothelial cell death. It was observed that in EC, the pharmacological inhibition of TRPM4 with 9-phenanthrol or glibenclamide protects the endothelium from Na^+^-dependent cell death [254].

LPS and ROS-induced cellular death was mediated by TRPM4. Also, using specific inhibitors such as 9-phenanthrol and glibenclamide, the H_2_O_2_-enhanced endothelial cell migration is diminished. Similar results were found using siRNA-TRPM4 [255]. Thus, it has been shown that TRPM4 overexpression eliminated H_2_O_2_ desensitization of TRPM4 in a dose-dependent manner and, at the same time, independently of PIP_2_. It was also determined by site-directed mutagenesis experiments that the C1093 residue was crucial for the loss of H_2_O_2_-mediated desensitization [256].

In the immune system, it has been described that TRPM4 regulates the migration of bone marrow-derived mast cells (BMMCs). BMMCs from knockout mice for TRPM4 (*trpm4*^−/−^) were not able to migrate after stimulation with dinitrophenylated human serum albumin or stem cell factor [257]. Thus, it has been reported that activated *trpm4*^−/−^ BMMCs produce a greater degranulation and release more histamine, leukotrienes and tumor necrosis factor-α (TNF-α) [258]. Furthermore, TRPM4 is crucial to the mobility of dendritic cells (DCs) but not to the maturation process [259]. On the other hand, the deletion of the *trpm4* gene has been reported to increase the mortality of mice in a model of sepsis induced by cecal ligation and puncture, also generating a systematic increase in LysC^+^ monocytes and the production of proinflammatory cytokines.

In T-lymphocytes, TRPM4-dependent depolarization modulates Ca^2+^ oscillations have been reported to increase the production of interleukin 2 (IL-2) [260]. Also, it has been described that TRPM4 regulates the function of Th1 and Th2 lymphocytes by the differential regulation of Ca^2+^ signaling and the location of the nuclear factor of activated T-cell cytoplasmic 1 (NFATc1) [261]. Furthermore, the alteration in Ca^2+^ homeostasis in *trpm4*^−/−^ macrophages downregulates the AKT signaling pathway, causing a decrease in the phagocytic activity of these cells. Conversely, no alteration in the function, distribution or mobilization of Ca^2+^ was found in *trpm4*^−/−^ neutrophils [262].

In recent years, evidence has emerged about the interaction of sulfonylurea receptor 1 (Sur1)-NC_Ca-ATP_ and TRPM4. The first to demonstrate a possible direct interaction between these proteins was Sala-Rabanal and collaborators, who, through electrophysiological and Förster resonance energy transfer (FRET) studies, determined that in COSm6 cells the interaction between SUR1 and TRPM4 was unlikely [263]. However, Woo and colleagues, using immunoprecipitation and FRET studies, determined that the de novo appearance of Sur1-Trpm4 heteromers existed after spinal cord injury in rats [264]. Subsequently, upregulation of the SUR1-TRPM4 heteromers was reported in human and rat brains with subarachnoid hemorrhage (SAH). Similar results were found by postmortem analysis of TRPM4 expression in infarcted human brains [265].

The role of TRPM4 in cardiac hypertrophy has also been reported by Guinamard and collaborators, who observed an increase in TRPM4 expression in spontaneously hypertensive rats, while no expression was detected in control rats [266]. This was corroborated by Demion and colleagues through morpho-functional analysis of the heart from *trpm4*^−/−^ mice, showing an increase in the wall thickness, the size of the left ventricular chamber and hypertrophy compared to *trpm4^+/+^* mice [267]. Furthermore, elimination of TRPM4 in mice cardiac muscle cells has been shown to result in hypertrophic growth after treatment with Angiotensin-II (AngII). At the same time, this was ratified by the increased expression of Regulator of Calcineurin1, atrial natriuretic peptide and α-actin [268]. In addition, pre-treatment with 9-phenanthrol in mouse hearts considerably decreased the area affected by infarction and restored the contractile function compared to control [269]. Following this, it has been shown that in *trpm4/trpm5^(^*^−/−*)*^ mice under β-adrenergic stimulation (Isoprenaline), there is no difference in the positive ionotropic response of papillary muscles as compared to the *trpm4*^−/−^ mouse. In a monocrotaline (MCT)-induced pressure load rat model, *trpm4*^−/−^ rats showed a significant increase in hypertrophy as compared to *trpm4^+/+^*. Also, a drastic decrease in TRPM4 protein levels in the right ventricle was determined in MCT-treated rats, while no changes in the left ventricle were observed in control animals [270].

The first reports of TRPM4 in the context of cancer showed that the inhibition of this channel in HeLa cells generated a decrease in cell proliferation [271]. In prostate cancer patient samples, it was reported that prostatic intraepithelial neoplasia and prostate cancer tissue present high TRPM4 levels compared to healthy tissue [272]. Furthermore, it has been observed that the knockdown of TRPM4 was able to decrease the migration of androgen-insensitive prostate cancer cell lines DU145 and PC3 but not its proliferation. Interestingly, a SOCE increase occurred after inhibition of TRPM4 by siRNA [272]. However, Sagredo and colleagues reported that the inhibition of TRPM4 leads to a decrease in proliferation in PC3 [271,273]. The same group evaluated the migration and invasion ability of PC3 cells inhibiting TRPM4 by short hairpin RNA and observed a decrease in the migration/invasion capacity [274]. On the other hand, overexpressing TRPM4 in LnCap cells increases Snail1 expression and the migration/invasion capacity [274]. Moreover, it was recently reported that the expression of TRPM4 is upregulated in breast cancer and associated with worse clinical-demographic parameters [275,276]. Indeed, it was reported that in prostate cancer, TRPM4 is regulated by microRNA-150 [277]. The same was also reported in colorectal and pancreatic cancer, opening a door to future anti-cancer therapies based on the inhibition of TRPM4 in signaling pathways associated with EMT [278,279]. Also, the inhibition of End Binding (EB) proteins-dependent anterograde trafficking of TRPM4 inhibits cell invasion in B16-F10 melanoma model [280]. As such, future anti-cancer therapies based on the inhibition of TRPM4 in signaling pathways seem promising.

In silico analysis of clinical samples has shown that the high expression of TRPM5 mRNA was correlated with a poor overall survival rate in patients with melanoma and gastric cancer but not in patients with ovarian, lung, breast or rectal cancer [281]. However, the same group noted that treating mouse tumors with triphenylphosphine oxide, a potent and selective TRPM5 inhibitor, showed a significant reduction in lung cancer metastasis [281,282]. On other hand, a hospital-based control case study of single nucleotide polymorphisms in gene regions related to immune responses associated polymorphisms in TRPM5 with altered susceptibility to developing childhood leukemia [283].

In 2010, Ketterer and collaborators, through a genome-wide association study, determined that there was a relationship between TRPM5 and the development of prediabetic phenotypes, including pancreatic β-cell dysfunction [284]. It has been reported that *trpm5*^–/–^ mice that underwent glucose tolerance tests maintained elevated glucose levels in the blood for over 1 h compared to WT mice. Furthermore, in pancreatic islets isolated from *trpm5*^–/–^ mice, arginine-induced hyperglycemia and insulin secretion were significantly reduced [285]. T*rpm5*^−/−^ mice are more resistant to carbohydrate-induced obesity and these mice had significantly lower increases in weight and fat mass when exposed to a high-fat diet and even developed less insulin resistance than control mice [286]. Interestingly, the *trpm5*^–/–^ mice were found to consume the same amount of calories when fed the high-fat diet and the high-fat diet plus a tasty chocolate ball-much the opposite of what happened with the WT mice, which considerably increased their caloric intake in the combined diet [287]. On the other hand, it has been shown in pancreatic islets isolated from a mouse model of type II diabetes that increased plasma insulin levels negatively regulate TRPM5 expression [288].

Recently, TRPM5 was reported to be involved in the response to high salt ingestion. Inhibition of TRPM5 results in a repulsive response to high salt, with reduced taste perception in the cortical field of the mice. Remarkably, this altered perception of bitter taste caused by high salt intake also existed in hypertensive patients with high salt intake [289]. Lastly, the same group demonstrated that by increasing the expression of TRPM5 in mice through the injection of bitter melon extract (BME) and cucurbitacin E (CuE), a major compound in BME, high salt-induced hypertension was reduced. Even long-term BME intake significantly improved aversion to high salt concentrations by upregulating TRPM5 expression and function, ultimately decreased excessive salt intake in mice and improved cardiovascular dysfunction induced by high salt and hypertension angiotensin II-induced [290]. These findings suggest that TRPM5 functions as a possible target to counteract the pathological effects of high-salt diets and hypertension.

Main TRPM4/TRPM5 endogenous and exogenous modulators, participation in diseases and physiological functions are listed in Table 3.

## 5. TRPM6-TRPM7

### 5.1. General Properties and Distribution

Transient Receptor Potential Melastatin 6 (TRPM6) and Transient Receptor Potential Melastatin 7 (TRPM7) are bifunctional proteins composed of an ion channel permeable to divalent cations, bound to the kinase domain [291]. The expression of these channels in human organs for TRPM6 is tissue-specific, mainly in the kidney, small intestine and colon, whereas TRPM7 has a ubiquitous expression [48,80].

Experiments using HEK-293 cells and the heterologous expression of human TRPM6 in CHOK1 cells have shown that TRPM6 and TRPM7 are non-selective channels permeable predominantly to Mg^2+^ and Ca^2+^. However, they are also permeable to a wide range of divalent cations including Ba^2+^, Mn^2+^, Sr^2+^, Cd^2+^, Ni^2+^ and Zn^2+^. In TRPM6, inward currents induced by Ca^2+^ and Mg^2+^ blocked monovalent inward currents with an affinity of 5.4 μM for Ca^2+^ and 3.4 μM for Mg^2+^ at −120 mV [292]. In HEK293 cells, TRPM6 selectivity and permeation to Mg^2+^ are regulated for the ^1028^GEIDVC^1033^ aminoacidic sequence from the pore region [293]. However, some authors suggest that the TRPM6 and TRPM7 selectivity pattern is modified depending on the tissue, physiological environment and diseases and becoming exclusively permeable to one of ion [292,294].

**Figure 5:**
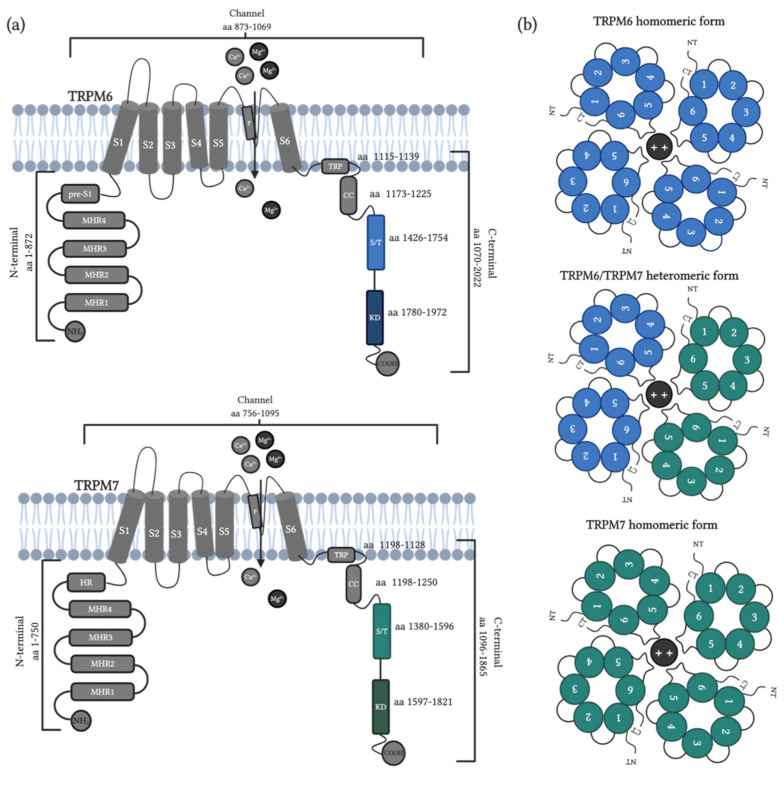
TRPM6 and TRPM7 structure and tetramer representation. (**a**) The N-terminus region is composed of four melastatin homology regions (MHR) and the pre-S1 (TRPM6) or homology region (HR, TRPM7) domain. The channel domain contains six transmembrane segments (S1–S6) and the pore-forming loop between the S5 and S6 segments. The C-terminus region is composed of TRP, coiled-coil (CC), Serine—Threonine (S/T) and kinase domains. aa: amino acid. (**b**) These channels form tetramers with four subunits in homomeric or heteromeric form forming a pore in the membrane and allows divalent cations to permeate. 1–6: transmembrane subunits, NT: N-terminus, CT: C-terminus. Created with BioRender.com.

The TRPM6 and TRPM7 structures are composed of the same three regions of the TRPM subfamily with MHR in the N-terminus, the channels between S1 to S6, the TRP domain for stabilization, CC for tetramerization and the S/T rich domain and α-kinase domain (KD) in the C-terminus (Figure 5a) [50,52,295,296,297]. Due to their structural similarities, TRPM6 and TRPM7 are closely related and can assemble heterotetrameric complexes after TRPM7 transphosphorylation by TRPM6 in a tissue-dependent manner (Figure 5b) [298,299]. The tetrameric channels assembled are composed of four homomeric subunits or two subunits of each protein and these arrangements modulate the pathophysiological role of TRPM6 and TRPM7 [292,300]. However, TRPM6/7 complex differs in some properties with respect to the TRPM6 and TRPM7 homomeric channels, including different permeability to Ni^2+^, differences in pore structure, specifically the number of negatively charged residues, 8 for TRPM6 and 7 for TRPM7, which produce variations in sensitivity to low pH in the three types of channels, and, finally, the conductance, which range between 82 to 84 pS for TRPM6, 40 to 105 pS for TRPM7 and 56.6 pS for TRPM6/7 heteromeric [67,292,296,301,302].

The kinase domain of these channels is a member of an atypical protein kinase family called α-kinase. These kinases have the particular substrate specificity in which they predominantly phosphorylate residues present in α-helices [303]. The kinase activity is related to its autophosphorylation activity in the S/T rich domain, which enables them to interact and phosphorylate other proteins [304]. In relation to this, it has been shown that autophosphorylation of the S/T rich domain plays an essential role in the control of the protein kinase activity of TRPM6 and TRPM7 by providing the access of the catalytic domain to the substrate [305].

### 5.2. Activation and Inhibition: Endogenous Modulators

The homomeric form of TRPM6 and TRPM7 but not the heteromeric from, remains inactive under physiological Mg^2+^ levels and has high responsiveness to cytosolic Mg^2+^ changes [306,307]. Nevertheless, the heteromeric form is active under basal conditions by the TRPM6 kinase domain activity and lacks sensibility to Mg^2+^ and Mg-ATP [308,309]. In particular, a bifunctional effect of adenosine triphosphate (ATP) has been demonstrated in TRPM7 activity, where initially it was reported that ATP activates TRPM7 by the chelation of Mg^2+^ [296]. However, other studies demonstrate that Mg-ATP, at millimolar concentrations, inhibits TRPM7 activity in a Mg^2+^-concentration-dependent manner [294].

TRPM6 and TRPM7 are endogenously inhibited by the loss of the interactions between the TRP domain and phosphatidylinositol bisphosphate (PIP_2_) due to its hydrolysis as a result of phospholipase C (PLC) activation. Xie and colleagues demonstrated that the interaction of TRPM6 and PIP_2_ is by the R1088 residue in the TRP domain but in TRPM7 the interaction its due to the histidine, arginine and lysine residues in the TRP domain with no specific residue identified yet [310].

Epidermal growth factor (EGF) increases the activity and expression of TRPM6 through ERK/AP-1 signaling, which is prevented by mutations performed in EGF [311,312,313]. It has also been shown that EGF-mediated TRPM6 activation is inhibited by anti-epidermal growth factor receptor drugs [314]. Similar effects occur with insulin, which increases the activity and recruitment of TRPM6, being abolished by diabetes treatment drugs [315,316]. Uromodulin modulates endocytosis and the abundance of TRPM6 in the plasmatic membrane of epithelial cells from the distal convoluted tubule, increasing TRPM6 activity during Mg^2+^ deficiency periods [317]. Other endogenous inhibitors of TRPM6 activity include ATP through its receptor P2X4 and hydrogen peroxide, which oxides M1755 in the TRPM6 kinase domain, inhibiting Mg^2+^ currents and being abolished by methionine sulfoxide reductase B1 [318,319].

Some studies have shown that TRPM6 expression and activity are modulated by different exogenous molecules. Metformin, a drug widely used to control sugar-blood concentration, inhibits TRPM6 expression in colon and kidney cells, which cause downregulated Mg^2+^ homeostasis [316]. Anti-epidermal growth factor receptor (EGFR) drugs used during cancer treatment cause TRPM6 upregulation and consequent hypomagnesemia in renal tubular epithelial NRK-52E cells. Nevertheless, GW-9662 and LE135 drugs can revert the reduction of Mg^2+^ reabsorption caused by anti-EGFR drugs [320]. Also, the activity of TRPM6 and heteromeric TRPM6/7 channel is modulated using a specific inhibitor, NS8593, which reduces the Mg^2+^ influx in kidney cells [299].

In HEK-293 cells, it has been shown that TRPM7 channel activity is sensitive to intracellular pH and Mg^2+^ and that this relation is biphasic in both cases. This means that the inhibition involves two separate sites of high and low Mg^2+^ affinity, when the intracellular solution contains a relative weak Mg^2+^ concentration [321]. In addition, in 2019, Inoue and colleagues demonstrate that reduced Mg^2+^ intracellular concentration in adipocytes and pre-adipocytes activates TRPM7-mediated Ca^2+^ influx [322].

In 2004, Takezawa and colleagues showed, in HEK293 cells, that the activation of muscarinic and β-adrenergic receptors modulates cyclic adenosine monophosphate (cAMP), which activates protein kinase A (PKA) and that this process is crucial for TRPM7 activation. Also, they showed that the TRPM7 endogenous kinase domain is not essential for channel gating. It remains unclear whether the relation between cAMP, PKA and TRPM7 is due to an interaction between these proteins or the phosphorylation of the S/T rich domain [323]. Nevertheless, in 2018, Broertjes and colleagues showed that TRPM7 Ca^2+^ currents in mouse neuroblastoma cells decreased when cAMP concentration increased and when the PKA catalytic domain was inhibited. This is due to the phosphorylation of S1269 residue near the CC domain of TRPM7 by PKA, inhibiting the gating of the channel and the Ca^2+^ influx stimulated by bradykinin [324]. Interestingly, it is known that bradykinins signal via two G-protein coupled receptors, B1 (BDKRB1) and B2 (BDKRB1). The activation of these receptors activates the PLC pathway and intracellular Ca^2+^ mobilization [325]. Thus, it is important to elucidate the mechanism by which bradykinin activates TRPM7 activity, taking into account that PLC protein, as we discussed before, inhibits the TRPM7 activity. Finally, in 2020, Sun and colleagues demonstrated TRPM7 activation by a β-adrenergic receptor agonist like isoproterenol and its activation regulates Mg^2+^ homeostasis in neuroblastoma cells [326]. The differences showed in these studies could be caused by differences in the methodology and different cell types used.

The oxidative stress is also related to the TRPM7 activation, in which an increased reactive oxygen species (ROS) extracellular concentration inhibits the TRPM7-dependent current in an Mg^2+^ but not ATP-dependent manner [327]. In contrast to these results, it has been shown that the ROS intracellular concentrations also modulate the activity of this channel, such that when the concentration of ROS is increased, TRPM7 expression and activity are also increased [328].

On the other hand, it has been shown that antipsychotic drugs, like aripiprazole, inhibit microglia inflammation by inhibiting the Ca^2+^ influx mediated by TRPM7 activity [329]. Also, 2-aminoethyl diphenyl borinate (2-APB) is widely used for the inhibition of TRPM7 activity, which exerts it activity by intracellular acidification [321]. Other drugs used are Sphingosine, fingolimod (FTY-720) and Carvacrol, which are immunosuppressants and anti-inflammatory drugs that prevent TRPM7 gating and ion influx [330,331]. Finally, under physiological conditions, it has been shown that TRPM7 activity is modulated by small molecules (i.e., Naltriben, Mibefradil, Sertraline, etc.), which activate this channel even under conditions of low PIP_2_ or decreased intracellular Mg^2+^ levels [332].

### 5.3. Physiological Function and Role in Diseases

It has been shown that both channels TRPM6 and TRPM7 are closely related to the maintaining of Mg^2+^ and Ca^2+^ homeostasis, which, in turn, is related to physiological process and disease development [333].

TRPM6’s contribution to Mg^2+^ homeostasis is essential in the kidney and small intestine [299], however, it has also been observed in mammary epithelial cells [334] and colon cells [335]. Further studies performed using TRPM6 heterologous expression in HEK923 cells and *Xenopus laevis* oocytes show that mutations in TRPM6 affect TRPM6/7 complex formation, inhibiting TRPM6 expression. It is hypothesized that these events strongly affect intestinal transport and renal Mg^2+^ reabsorption [336,337].

Likewise, TRPM6 participates in other relevant physiological processes, including early embryo development, where TRPM6 is essential for survival and its study using murine models shows that its lack is lethal [338,339]. Further study of TRPM6’s role in *Xenopus laevis* embryogenesis has shown that TRPM6 expression begins in gastrulation and its higher expression levels occur in the neurulation stage. Abnormalities in TRPM6 function caused by microinjection of an antisense morpholino oligonucleotide targeting TRPM6 induced defects in gastrulation and neural tube closure and some embryos exhibited alterations in head, eye and brain size. Finally, experiments using TRPM6 depletion showed that a lack of TRPM6 specifically affects pole cell radial intercalation, leading to defects in blastopore closure during the neural tube closure process [340].

Deletion of TRPM7 channel was lethal in DT-40 B lymphocyte cells [294]. Also, in this cell type, it has been shown that this channel regulates the Ca^2+^ homeostasis through the regulation of Store-Operated Calcium Entry (SOCE) by its kinase activity, where blocking this channel results in a significant downregulation of the refill Ca^2+^ stores process [341].

In the membrane of acetylcholine-secreting synaptic vesicles from sympathetic neurons, TRPM7 channel activity is crucial for spontaneous vesicle fusion [342]. In adipocytes, during physiological conditions, TRPM7 mediates the influx of Ca^2+^ depending on intracellular Mg^2+^ concentration and the actions of different inhibitors like 2-aminethoxydiphenyl borate (2-APB), hydrogen peroxide (H_2_O_2_) and other drugs, which inhibit the TRPM7-mediated Ca^2+^ current [322]. In endothelial cells (EC), it has been demonstrated that TRPM7 is related to the growth and proliferation process by the extracellular signal-regulated kinase (ERK) signaling pathway, where the inhibition of TRPM7 activity using 2-APB, Mg-ATP, Gd^3+^ or specific small interference RNA (siRNA) decreases these processes by the reduction of the expression and amplitude of the TRPM7-like current [343].

In a recent study, Mittermeier and colleagues (2019) using genetic and biophysical approaches explored the role of TRPM7 beyond its known role in Ca^2+^ balance, establishing a key role of TRPM7 channel activity in maintaining Zn^2+^, Mg^2+^ and Ca^2+^. The authors demonstrated TRPM7 importance in organismal mineral homeostasis, demonstrating that intestinal and renal TRPM7 regulation of these minerals is necessary for postnatal growing and survival [344].

In animal models, Ryazanova and colleagues showed that kinase domain deletion in homozygous mice generates embryonic lethality but the heterozygous mice are viable and show hypomagnesemia with reduced intestinal Mg^2+^ absorption due to reduced TRPM7 currents provoking decreased levels of Mg^2+^ in plasma, bones, erythrocytes and urine in adult mice. These results indicate that this channel has a fundamental and importantly nonredundant role in cellular physiology and development [345].

Mg^2+^ participates in several enzymatic reactions. Therefore, metabolic abnormalities that affect its transport through TRPM6 generate systemic alterations and pathologies with considerable gravity [346,347]. Hypomagnesemia with secondary hypocalcemia (HSH) is an autosomal recessive condition deeply studied, characterized by neuromuscular disorders due to Mg^2+^ deficiency [348] and genetic studies carried out in patients have identified several mutations in TRPM6 causing the disease [349,350].

Genetic studies carried out on infant patients associate two genetic variations of TRPM6 with embryo development defects. First is the TRPM6 reference SNP (single nucleotide polymorphism) rs3750425 (G>A; V1393I), associated with meningomyelocele, a tube neural disorder that affects osteogenesis and causes a spina bifida-like phenotype and reduced Mg^2+^ levels in serum [351] and the homozygous mutation in exon 19 of TRPM6 gene (Chr9: 77407598; C>T; c. 2480G>A) that results in a stop codon and premature truncation of the TRPM6 protein, causing reversible epileptic childhood encephalopathy [352]. Moreover, the TRPM6 rs2274924 in Chinese patients confers susceptibility to post-stroke epilepsy development, also affecting Mg^2+^ serum levels [353].

TRPM6 disorders have also been associated with clinical complications derived from chronic diseases such as diabetes [354,355], where TRPM6 V1393I and TRPM6 K1584E polymorphisms present in pregnant women with insulin resistance generate high susceptibility to developing gestational diabetes mellitus or diabetes mellitus type 2, possibly by the underlying defects in Mg^2+^ transport that affect insulin receptor sensitivity [315]. In a hypertension murine model, aldosterone infusion affected TRPM6 expression in the plasmatic membrane, triggering hypomagnesemia and worsening hypertension-induced renal injury. Correction in Mg^2+^ levels through supplementation diminished aldosterone-induced blood pressure increase, renal fibrosis and oxidative stress due to increased TRPM6 expression and activity, suggesting a close interaction between TRPM6 activity and aldosterone effects on cardiovascular and renal function [356]. Finally, genetic profile analysis in cancer samples targeted TRPM6 gene into 10 hub genes for colorectal cancer development and the regulation of its expression by the miRNA Hsa-let-7f-1 is key for patient survival [357].

The TRPM7 function and dysfunction are closely related to the pathogenesis of multiple cancers. Several studies of prostate cancer cells have shown that the Ca^2+^/Mg^2+^ ratio has an important role in the initiation and progression of this type of cancer, added to the importance of hypoxia in TRPM7 activity and its role in the epithelial-mesenchymal transition and cell migration of cancer cells due to TRPM7-HIF-1α-Anexin-1 signaling axis activation [358,359]. TRPM7 but no TRPM6 expression, it is also important in the predisposition of patients with inflammatory bowel disease to colorectal cancer (CRC) and the progression of CRC, where it has been shown that TRPM7 expression is related to tumor infiltration, lymph node metastasis, distant metastasis and advanced clinical stage in patients [360,361].

Related to this, lymphocytes of a mice model with inactive TRPM7 kinase showed decreased pro-inflammatory cytokine secretion, less intraepithelial infiltration and retention, reduced differentiation to Th17 lymphocytes and a protective effect to acute graft-versus-host disease [362].

TRPM7 activity has also been related to delayed neuronal death (DND) after ischemic injury, where the suppression of this channel in vitro and in vivo in the CA1 neuron of adult rats using siRNA packaged in an adeno-associated virus inhibits ischemic DND, improves recovery in surviving TRPM7-deficient neurons and prevents the loss of memory functions [363].

Our group previously demonstrated that during an endotoxic condition, a model of study of sepsis in vitro, EC suffers a transition to fibroblast by the activation of toll-like receptor 4 (TLR4) and increased ROS production through NAD(P)H activation [364]. TRPM7 plays an important role in this endotoxin-induced extracellular fibrosis increase by means of the Ca^2+^ influx, expression of extracellular matrix (ECM) protein like type III collagen and fibronectin, fibrotic markers like a-smooth muscle actin and fibroblast-specific protein 1 and decreased EC markers like platelet endothelial cell adhesion molecule and vascular endothelial cadherin, which is a classic phenotype of an endothelial-to-mesenchymal transition mechanism in which EC turns into activated fibroblast [365]. Furthermore, it has been shown that TRPM7 is also involved in the cell migration pattern of EC during endotoxic conditions, in which both the expression inhibition by siRNA and pharmacological blocking of TRPM7 activation, inhibit EC migration [366]. We recently demonstrated that the role of TRPM7 in the progression of endotoxemia is that the channel expression and activity increase the secretion of pro-inflammatory cytokines like tumor necrosis factor-a (TNF-α), IL-1β, IL-6 and IL-12 without changing the anti-inflammatory cytokine production. Here, we also showed that TRPM7 is related to metabolic dysfunction, pancreatitis, cardiac muscle damage and muscle mass wasted in rats [367]. Additionally, we demonstrated that TRPM7 expression and activity are related to renal vascular hyperpermeability, kidney dysfunction and increased mortality in rats with endotoxemia [61,368]. TRPM7 expression and levels of TNF-α, IL-6 and IL-1β are increased in the serum of patients with sepsis diagnosis and those patients with sepsis and a high expression of TRPM7 have a decreased survival rate in comparison to patients with a low expression of TRPM7 [369]. These findings support the hypothesis that TRPM7 is closely related to systemic symptoms, organ dysfunction and mortality during endotoxemia, suggesting that this channel could be a good target for the treatment of endotoxemia, sepsis and other inflammatory diseases.

In TRPM7-deficient mice, Schappe and colleagues demonstrated that TRPM7 is necessary for endotoxin-induced macrophage activation. In addition, they showed that TRPM7 participates in endotoxin signaling modulating the Ca^2+^ influx necessary for TRL4 endocytosis and transcriptional activity in LPS-induced peritonitis [370].

It is also demonstrated that ROS production during endotoxic conditions increases intracellular Ca^2+^ and cell death in primary hippocampal and differentiated PC12 neurons. However, TRPM7 expression inhibition by siRNA shows a protective effect in these cell types against an endotoxic stimulus, suggesting a pivotal role of TRPM7 in neuronal cell death during endotoxic conditions [371]

Also, it has been shown that TRPM6 and TRPM7 modulation at the same time modulate the response to diseases like sepsis. Specifically, a study of septic Wistar rats showed a protective effect of salvianolic acid B (SA-B) on acute lung injury (ALI), decreasing TRPM6 and TRPM7 expression, which provokes a consequent downregulation of pro-inflammatory cytokines in lung tissues and reduced mortality [359].

TRPM7 plays a pivotal role in the progression of nephropathy with kidney fibrosis, where it was shown that TRPM7 is upregulated during renal damage in a unilateral ureteral obstruction mouse model [372]. Also, it has been demonstrated that TRPM7 mediates neuronal cell death during neonatal hypoxic-ischemic brain injury, a model of hypoxic-ischemic encephalopathy, by the regulation of calcium/calmodulin-dependent protein kinase II, calcineurin p38 and cofilin cascade, where the inhibition of TRPM7 using waixenicin A reduces brain injury and improves the short- and long-term functional outcomes [373].

Finally, Genetic variation in *trpm7* gen is related to channel disfunction in an unexplained stillbirth population improving a fatal arrhythmia process in utero. Related to this, the most important TRPM7 variants were pG179V and pT860M, which led to a marked reduction in the ion channel expression [374].

Main TRPM6/TRPM7 endogenous and exogenous modulators, participation in diseases and physiological functions are listed in Table 4.

## 6. Conclusions

TRPM ion channels have emerged as a group of proteins with huge biomedical potential because of their participation in several physiological functions, as well as in several human pathologies. The latest findings have shown us the molecular basis of channelopathies induced by TRPM ion channel malfunction. For that reason, medical practice and the pharmaceutical industry have focused on them as novel targets for diagnostic, treatment and drug design and therapeutic approaches. However, significant basic and clinical research must be performed to respond to questions regarding channelopathies induced by TRPM ion channels that still remain to be resolved.

## Figures and Tables

**Table 1:** Molecules modulating the activity of TRPM1 and TRPM3, participation in physiological processes and pathologies.

Channel	Modulator	Inhibition or Activation ^1^	Function	Disease, Affection or Objective of Study ^2^	Model of Study	Ref.
TRPM1	Hexamethylene bisacetamide (HMBA)	+	Melanocyte differentiation	Melanoma	Pigmented melanoma cells	[132]
	Pregnolone sulphate	+	Ca^2+^ currents	Insulin secretion	HEK-293 cells, Pancreatic	[102]
	Capsaicin	+	Field potential	Retinal synaptic plasticity	Mouse model	[133]
	Extracellular Zn^2+^	-	Ca^2+^ currents	Insulin secretion	HEK-293 cells, Pancreatic Islets	[102]
	Voriconazole	-	ON-bipolar cells in mouse retina	congenital stationary night blindness	ON-bipolar cells of mouse retina	[134]
	NED-180	-	Ca^2+^ currents	Melanocyte	HEK-293 cells	[135]
TRPM3	Low intracellular Ca^2+^	+	Ca^2+^ entry	PC	HEK-293 cells	[62]
	Heat (40 °C)	+	Ca^2+^ currents	PC	HEK-293 Neurons (DRG, TGN)	[116]
	Hypotonicity	+	Channel activation	Kidney function	HEK-293 cells	[66]
	Derythro-sphingosine, *N*,*N*-dimethyl-d-erythro-sphingosine	+	Ca^2+^ currents	Channel activation	HEK-293 cells	[85]
	Pregonolone sulphate	+	Mg^2+^ currents	Neuro endotoxin- induces loss of neuron cells	HEK-293 Pancreatic β-cells	[136]
	Pregnolone	+	Ca^2+^ currents	Nociception	HEK-293 Neurons (DRG, TGN), mouse models.	[137]
	Dehydroepiandrosterone (DHEA)	+	Ca^2+^ currents	Nociception	HEK-293 Neurons (DRG, TGN), mouse models.	[137]
	Epiallopregnanolone sulfate	+	Ca^2+^ currents	Insulin secretion	HEK-293, Pancreatic islets	[102]
	Intracellular Mg2+	-	Ca^2+^ currents	Insulin secretion	Pancreatic β-cells	[64]
	La^3+^	-	Ca^2+^ currents	PC	HEK-293 Neurons (DRG, TGN)	[108]
	Gd^3+^	-	Partial inhibition of Ca^2+^ entry	Channel activation/inhibition	HEK-293 cells	[107]
	Clotrimazole	+	Ca^2+^ currents	Anoxic neural damage	HEK-293 Neurons (DRG, TGN), pancreatic islet.	[108]
	Nifedipine	-	Ca^2+^, Mg^2+^ currents and mortality	PC	HEK-293 Pancreatic b-cells	[136]
	CIM0216	+	Ca^2+^	Pain and insulin secretion	HEK-293 Neurons (DRG, TGN), pancreatic islet.	[108]
	Progesterone	-	Ca^2+^ current	Cardiovascular function	HEK-293 and Vascular smooth muscle cells	[138]
	Cholesterol	-	Ca^2+^ current	Cardiovascular function	Vascular smooth muscle cells	[97]
	Fenamates	-	Ca^2+^ current and insulin secretion	Pancreatic function	HEK-293, Insulin secreting cells	[139]
	Fenamates	-	Ca^2+^ current and insulin secretion	Pancreatic function	HEK-293, Insulin secreting cells	[139]
	Citrus fruit flavonoids	-	Intracellular Ca^2+^	Nociception	HEK293, Neurons (DRG)	[140]

^1^: activation (+) and inhibition (-); ^2^: physiological conditions (PC).

**Table 2:** Molecules modulating the activity of TRPM2 and TRPM8, participation in physiological processes and pathologies.

Channel	Modulator	Inhibition or Activation ^1^	Function	Disease, Affection or Objective of Study ^2^	Model of Study	Ref.
TRPM2	ADPR	+	Ca^2+^ currents	PC	HEK293 cells and Jurkat T lymphocytes	[160,219]
	ROS	+	Cell death	PC	HEK293 cells	[187,220]
	pH	-	Ca^2+^ currents	PC	HEK293 cells	[221,222]
	Curcumin	-	Production of ROS	Hepatic damage	Hooded Wistar rat	[168]
	2-APB	-	Ca^2+^ currents	PC	HEK293 cells	[169]
	Flufenamic acid	-	Ca^2+^ currents	PC	HEK293 cells	[170]
	Imidazoles clotrim-azole and econazole	-	Ca^2+^ currents	PC	HEK293 and CRI-G1	[171]
	Cacospongiasp. OrganicExtract	-	Ca^2+^ currents	PC	HEK293 cells	[175]
	PJ34 and DPQ		Inhibition of poly (ADP-ribose) polymerase	PC	HEK293 cells	[176]
	TRPM2-knockou and siRNA	-	Ca^2+^ currents and JNK pathway activation	Renal fibrosis	C57BL/6J mice and HK-2	[189]
	shRNA	-	Production of inflammatory mediators and decreased apoptosis-related protein expressions	Sepsis	Human primary monocytes	[223]
	siRNA and Trpm2-deficient mice	-	CXCL2 expression, neutrophil infiltration	Inflammation	Mouse monocytes	[224]
	TRPM2-KO	-	Neuronal toxicity and memory impairment	β-amyloid-mediated neuronal toxicity	APP/PS1 mice	[191]
	8-Br-cADPR	-	Inhibition of renal ischemia–reperfusion injury	renal ischemia–reperfusion injury	Wistar rat	[193]
TRPM8	Cold and Menthol	+	Ca^2+^ currents	PC	CHO-K1/FRT cells	[141,177]
	Icilin	+	Ca^2+^ currents	PC	Trigeminal ganglia from newborn Sprague-Dawley rats	[154,177]
	Menthol derivates CPS-368, CPS-369, CPS-125, WS-5 and WS-12	+	Ca^2+^ currents	PC	Xenopus oocyte system and channel activity assayed	[180]
	2-(1*H*-Indol-3-yl)-*N*-(4-phenoxybenzyl)ethanamine	+	Ca^2+^ currents	PC	HEK293	[182]
	PIP_2_	+	Ca^2+^ currents	PC	HEK293 and Xenopusoocytes	[225]
	*N*,*N*-Dibenzyl-2-(1*H*-indol-3-yl)ethanamine	-	Ca^2+^ currents	PC	HEK293	[182]
	DFL23693 and DFL23448	-	Ca^2+^ currents	Induction of orofacial and neuropathic pain	HEK293, Sprague-Dawley rats	[183]
	IGM-18	-	Ca^2+^ currents and pain reduction	Induction of orofacial and neuropathic pain	Sprague-Dawley rats	[184,226]
	IGM-5	+	Increase in body temperature	Induction of orofacial and neuropathic pain	Sprague-Dawley rats	[184,226]
	TRPM8-KO	-	Sensing temperature	Thermosensation	C57BL/6 mice	[212,214]

^1^: activation (+) and inhibition (-); ^2^: physiological conditions (PC).

**Table 3:** Molecules modulating the activity of TRPM4 and TRPM5, participation in physiological processes and pathologies.

Channel	Modulator	Inhibition or Activation ^1^	Function	Disease, Affection or Objective of Study ^2^	Model of Study	Ref.
TRPM4	PLC > PIP_2_ > IP_3_ > Ca^2+^ rise	+	Channel activation	PC	293T, HEK 293 and rosetta cells	[41,228,230,232,237]
	ATP PKC	- +	Ca^2+^ sensitivity	PC	HEK 293 cells	[228]
	ADP, ATP, AMP and adenosine	-	Channel activation	PC	HEK 293 cells	[232,238]
	CAM	-	Ca^2+^ sensitivity, activation and shift the voltage dependence of activation	PC	HEK293 cells	[235]
	Heat	+	Temperature- dependent activation curve	Thermal sensitivity of sweet taste	HEK293 cells	[241]
	Flufenamic acid and spermine	-	Channel activation	PC	HEK 293 cells	[232,243]
	9-phenanthrol	-	Channel activity	PC	HEK293 cells	[244,245]
	Glibenclamide	-	Channel current	PC	HEK293 cells	[246]
	CBA	-	Channel current	PC	HEK293 cells	[246,247]
TRPM5	PLC > PIP_2_ > Ca^2+^ rise	+ -	Channel activation, desensitization and sensibilization	PC	CHO-K1 or HEK-293 M1, 293T cells	[41,230,232,236]
	ATP	+	Channel activation	PC	293T cells	[41,230]
	Heat	+	Temperature- dependent activation curve	Thermal sensitivity of sweet taste	HEK293 cells	[241]
	pH 6.2–5.9	-	Channel activation	Taste transduction	HEK293 cells	[242]
	Flufenamic acid and spermine	-	Channel activation	PC	HEK 293 cells	[232,243]

^1^: activation (+) and inhibition (-); ^2^: physiological conditions (PC).

**Table 4:** Molecules modulating the activity of TRPM6 and TRPM7, participation in physiological processes and pathologies.

Channel	Modulator	Inhibition or Activation ^1^	Function	Disease, Affection or Objective of Study ^2^	Model of Study	Ref.
TRPM6	PIP_2_ and PLC activity	+	Mg^2+^ currents and homeostasis	HSH	HEK-293 cells	[310]
	Cytosolic Mg^2+^ levels	-	Mg^2+^ currents	Hypomagnesemia	Murine models	[306]
	EGF/ERK/AP-1 signaling	+	Mg^2+^ transport and homeostasis	PC	HEK-293 cells	[311,312]
	Insulin	+	Mg^2+^ homeostasis	Glucose tolerance during pregnancy	Murine model	[315]
	Metformin	-	Mg^2+^ homeostasis and TRPM6 expression	Type 2 Diabetes	HEK-293 and hCaco-2 colon cells	[316]
	Uromodulin	+	Renal Mg^2+^ homeostasis	Low-magnesium diet	Murine model	[317]
	P_2_X_4_ receptor activity	-	Mg^2+^ transport and homeostasis	PC	Murine model	[318]
	MsrB1	+	Renal Mg^2+^ homeostasis	Oxidative Stress	HEK-293 cells	[319]
	Pharmacological activity	-	Intestinal and renal Mg^2+^ absorption	PC	HEK-293 cells	[299]
	Cytosolic Mg^2+^ levels	+	Mg^2+^ currents	Low-magnesium diet	Mammary epithelial cells	[334]
	Pharmacological activity	-	Mg^2+^ currents	PC	Human colon cells	[335]
	TRPM6 depletion	-	Neural tube closure	Embryogenesis	*Xenopus laevis*	[340]
	Homozygous deletion	-	Mg^2+^ homeostasis	Embryonic development	Murine models	[336,338]
	TRPM6 mutation	-	Intestinal and kidney Mg^2+^ homeostasis	HSH	Human DNA	[349]
	TRPM6 mutation	-	Mg^2+^ homeostasis	Hypomagnesemia	Baby human	[350]
	TRPM6 polymorphisms	-	Mg^2+^ and Ca^2+^ serological concentration	Meningomyelocele	Human serum	[351]
	TRPM6 polymorphisms	-	Mg^2+^ serological concentration	Post-stroke Epilepsy	Human serum	[353]
	Colorectal cancer	-	TRPM6 expression	Colorectal cancer	Colon cancer tissue	[357]
	Anti-EGFR drugs; GW-9662 and LE135	+/-	TRPM6 expression and kidney reabsorption	Cancer treatment	NRK-52E cells	[320]
	SA-B	-	TRPM6 expression and pro-inflammatory cytokines secretion	ALI during sepsis	Rat model	[375]
TRPM7	Intracellular pH and Mg^2+^	-	Ca^2+^ currents	PC	HEK-293 and Jurkat T cells	[321]
	cAMP/PKA signaling	+	Ca^2+^ currents	PC	HEK-293 cells	[323]
	Isoproterenol	+	Mg^2+^ currents	Neuro endotoxin-induces loss of neuron cells	Neuroblastoma SHSY-5Y cells	[326]
	ROS	+	Ca^2+^ currents	Anoxic neural damage	HEK-293 and Cortical neurons	[328]
	ATP	+	Ca^2+^ currents	PC	CHO-K1 cells	[296]
	Mg-ATP and Mg-GTP	-	Ca^2+^, Mg^2+^ currents and mortality	PC	HEK-293 and DT-40 B lymphocytes	[294]
	S1296 residue from TRPM7 TRP domain by PKA	-	Ca^2+^ currents	PC	Neuroblastoma cells	[324]
	Reduced Mg^2+^ intracellular, H_2_O_2_ and pharmacological activity	+	Ca^2+^ and Mg^2+^ currents	Function in adipocytes	Adipocytes	[322]
	Kinase domain mutation and H_2_O_2_ response	-	Intracellular levels and Mg^2+^ currents	Oxidative stress	HEK-293 cells and murine models	[327]
	Pharmacological activity	+	Ca^2+^ currents by SOCE and Proliferation	PC	DT-40 B lymphocytes	[341]
	Mg-ATP, Gd^3+^ and pharmacological activity	-	Growth and Proliferation	Low Mg^2+^ and Ca^2+^ environment	EC	[343]
	Kinase domain deficient TRPM7 channel	-	Mg^2+^ homeostasis	Embryonic development	Murine model	[345]
	Bradykinin	+	Upregulation of proinflammatory proteins and Mg^2+^ currents	Proinflammatory signaling	Smooth muscle cells	[356,376]
	PIP_2_ hydrolysis and PLC activity	-	Ca^2+^ and Mg^2+^ currents	PC	HEK-293T and CHO-K1 cells	[377]
	LPS	+	Ca^2+^ current and endothelial fibrosis	Endotoxic Condition	EC	[364,365]
	LPS	+	Endothelial migration	Endotoxic condition	EC	[366]
	LPS in plasma	+	Secretion of pro-inflammatory cytokines, metabolic dysfunction and organ failure	Endotoxemia	Rat model	[367]
	LPS in plasma	+	Vascular permeability, kidney damage and increased mortality	Endotoxemia	Rat model	[61]
	TRPM7 polymorphisms	-	Mg^2+^ currents	Arrhythmia associated to unexplained stillbirth	HEK-293, CHO-K1 and hiPSC-derived cardiomyocytes cells	[374]
	Aripiprazole	-	Ca^2+^ current	Inflammation	Microglia primary culture from mice models	[329]
	Carvacrol, FTY720, Sphingosine, 2-APB	-	Ca^2+^, Mg^2+^ currents	PC	HEK-293 and Jurkat T lymphocytes	[321,330,331]
	Small drug-like compounds	+	Ca^2+^ current	PC	HEK-293 cells and murine models	[332]
	LPS in plasma	+	TLR4 endocytosis, Ca^2+^ current, NF-κB and IRF3 transcription and translocation	Endotoxemia	Mouse model	[370]
	LPS	+	Neuronal cell death	Endotoxic conditions	Primary hippocampal and PC12 neurons	[371]
	Inactive TRPM7-kinase	-	Proinflammatory cytokines secretion, lymphocytes differentiation to Th17	PC and acute graft-versus-host disease	Mouse model	[362]
	siRNA	-	Membrane voltage and vesicle fusion	PC	PC12 cells	[342]
	siRNA	-	Neuron survival and memory retention	DND after ischemic injury	CA1 neurons and rat model	[363]
	Ca^2+^/Mg^2+^ external ratio and hypoxia	+	Ca^2+^ current, cell proliferation, HIF-1α accumulation and RACK1 phosphorylation	Prostate cancer	Prostate cancer cells	[358,359]
	TRPM7 expression	+	Tumor development, morphology and proliferation	Inflammatory bowel disease and CRC	Tumor tissue and colorectal cancer cells	[360,361]
	Fibrosis stimulus	+	Kidney atrophy, tubular formation and cell proliferation	Nephropathy	Unilateral ureteral obstruction mouse model	[372]
	Hypoxic and ischemic physiopathology	+	Protein expression, brain injury and outcome score	Hypoxic-ischemic encephalopathy	Hypoxic-ischemic brain cell death model	[373]
	SA-B	-	TRPM7 expression and pro-inflammatory cytokines secretion	ALI during sepsis	Rat model	[375]

^1^: activation (+) and inhibition (-); ^2^: physiological conditions (PC).
